# Barrier-to-Autointegration Factor Proteome Reveals Chromatin-Regulatory Partners

**DOI:** 10.1371/journal.pone.0007050

**Published:** 2009-09-16

**Authors:** Rocío Montes de Oca, Christopher J. Shoemaker, Marjan Gucek, Robert N. Cole, Katherine L. Wilson

**Affiliations:** 1 Department of Cell Biology, Johns Hopkins University School of Medicine, Baltimore, Maryland, United States of America; 2 Mass Spectrometry and Proteomics Facility, IBBS, Johns Hopkins University School of Medicine, Baltimore, Maryland, United States of America; National Institute on Aging, United States of America

## Abstract

Nuclear lamin filaments and associated proteins form a nucleoskeletal (“lamina”) network required for transcription, replication, chromatin organization and epigenetic regulation in metazoans. Lamina defects cause human disease (“laminopathies”) and are linked to aging. Barrier-to-autointegration factor (BAF) is a mobile and essential component of the nuclear lamina that binds directly to histones, lamins and LEM-domain proteins, including the inner nuclear membrane protein emerin, and has roles in chromatin structure, mitosis and gene regulation. To understand BAF's mechanisms of action, BAF associated proteins were affinity-purified from HeLa cell nuclear lysates using BAF-conjugated beads, and identified by tandem mass spectrometry or independently identified and quantified using the iTRAQ method. We recovered A- and B-type lamins and core histones, all known to bind BAF directly, plus four human transcription factors (Requiem, NonO, p15, LEDGF), disease-linked proteins (e.g., Huntingtin, Treacle) and several proteins and enzymes that regulate chromatin. Association with endogenous BAF was independently validated by co-immunoprecipitation from HeLa cells for seven candidates including Requiem, poly(ADP-ribose) polymerase 1 (PARP1), retinoblastoma binding protein 4 (RBBP4), damage-specific DNA binding protein 1 (DDB1) and DDB2. Interestingly, endogenous BAF and emerin each associated with DDB2 and CUL4A in a UV- and time-dependent manner, suggesting BAF and emerin have dynamic roles in genome integrity and might help couple DNA damage responses to the nuclear lamina network. We conclude this proteome is a rich source of candidate partners for BAF and potentially also A- and B-type lamins, which may reveal how chromatin regulation and genome integrity are linked to nuclear structure.

## Introduction

The nuclear envelope and lamin filament networks tether chromatin and influence chromatin organization and gene expression at several levels [1], [2], through mechanisms that remain obscure. A- and B-type lamins form separate networks of nuclear intermediate filaments, which are anchored to inner nuclear membrane (INM) proteins but also ramify throughout the nuclear interior [3], [4]. Lamins interact with chromatin and support most nuclear activities including replication, transcription and DNA damage repair [5]–[8]. Mutations in A-type lamins or other lamina components cause more than 15 human diseases (“laminopathies”) including Emery-Dreifuss muscular dystrophy and accelerated aging syndromes (i.e., atypical Werner, Hutchinson-Gilford Progeria Syndrome) [9], [10]. Cells expressing mutant lamins have compromised nuclear integrity. For example, in Hutchinson-Gilford progeria cells the mutated lamin A precursor (“progerin”) disrupts the levels or distribution of wildtype lamins, LEM-domain proteins, histone-modifying enzymes and histone posttranslational modifications, and also responses to DNA damage [5], [7], [11]. Another protein with essential roles in nuclear integrity is BAF, a conserved direct partner for lamins [12], [13] and the LEM-domain family of nuclear proteins. All characterized members of this family, including LAP2β, Emerin, MAN1 and Lem2/NET25 at the INM, and LAP2α in the nuclear interior, bind BAF via their LEM-domain, and also bind directly to lamins (reviewed by [14]). Studies in *Caenorhabditis elegans* and mammalian cells showed that BAF is required for the integrity of the nuclear lamina network, and in combination with LEM-domain proteins and lamins has interdependent structural roles during mitosis and nuclear assembly [13], [15]–[18].

BAF is an abundant, highly conserved and essential protein in metazoans (reviewed by [13], [19]). BAF is small (89 residues; 10 kD) but forms stable obligate dimers that can bind two dsDNA molecules [20]–[23]. BAF binds the dsDNA phosphate backbone, and thus has no DNA sequence specificity [23], [24]. BAF dimers (henceforth, “BAF”) diffuse rapidly within living cell nuclei [25], [26]. However, BAF can be immobile at specific times and locations, including anaphase when BAF localizes at telomeres, and telophase when BAF concentrates at “core regions” of decondensing chromosomes to mediate early steps in nuclear envelope and lamina assembly [18], [27]. In addition, BAF binds directly to nucleosome-exposed core and tail regions of histone H3 and linker histone H1 [28], and is required for chromatin structure and function in interphase cells [26], [29], and cell cycle progression [30], [31]. However, the mechanisms by which BAF influences mitosis, chromatin structure, gene expression [32], and other activities including retrovirus infection [33], [34] are not understood.

We hypothesized that BAF influences these various activities by associating with novel partners. To discover such partners, we used affinity purification and mass spectrometry methods to identify proteins from HeLa cell nuclear extracts that specifically associated with recombinant purified human BAF dimers. This strategy yielded several expected partners (lamins, core histones and LEM-domain protein LAP2), plus many novel candidate interactors including four human transcription factors and several proteins that modify or repair chromatin. Association with endogenous BAF was confirmed *in vivo* for seven of 11 candidates tested. We further report that BAF and its nuclear membrane partner emerin dynamically interact with DNA damage response proteins of the CUL4-DDB-ROC1 complex, suggesting novel roles for BAF and emerin in genome integrity.

## Results

**Figure 1:**
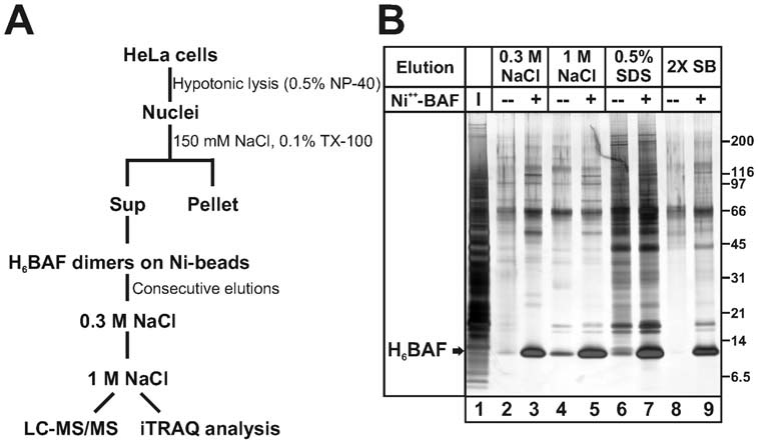
Affinity purification of BAF-associated proteins from HeLa cell nuclear lysates. (A) Schematic representation of the strategy used to purify BAF-associated proteins from HeLa cell nuclear lysates. (B) Silver-stained SDS-PAGE of input (I) proteins and proteins eluted from either Ni-beads alone (−) or Ni-BAF beads (+), by sequential elution with 0.3 M NaCl, then 1 M NaCl, then 0.5% SDS and finally 2X-SB. Shown is a representative gel (n = 3), with 10% of each sample loaded per lane. Molecular weight markers are indicated.

To identify proteins that potentially associate with BAF we affinity-purified proteins from HeLa nuclear extracts using recombinant purified His-tagged human BAF (H_6_BAF) dimers bound to Ni^++^-agarose beads, with agarose beads alone as controls (see Methods). Bound proteins were eluted sequentially with buffer containing 0.3 M NaCl to remove proteins of moderate affinity, then with 1 M NaCl to elute higher-affinity partners (Figure 1A). An aliquot (10%) of each eluate was resolved by SDS-PAGE and silver-stained for qualitative analysis. Reproducible patterns of BAF-associated bands were seen (Figure 1B; n = 3). The remaining eluates (90%) from a single experiment were digested with trypsin in solution and all detectable constituent proteins were identified by liquid chromatography-coupled tandem mass spectrometry (LC-MS/MS). Data were analyzed using an in-house Mascot server and Scaffold software, resulting in over 70 significant, unique, and non-redundant protein “hits” (Table S1). Proteins identified by Scaffold with at least 89% confidence in the 0.3 M or 1 M NaCl eluates from BAF-beads, and not detected in bead-only controls, are listed in Table 1. Table 2 lists selected proteins that were detected with at least 89% confidence in a BAF-bead eluate(s) and also in a bead-only control(s). A few proteins appear in both tables based on peptides that fit each criterion. The full (100 megabyte) searchable Scaffold dataset is available upon request. We also scaled up sample preparation, and used isobaric tagging for relative and absolute quantitation (iTRAQ) reagents to independently identify and quantify proteins that were specifically enriched on BAF-beads, relative to bead-only controls. For iTRAQ analysis, the four samples were each labeled with a different iTRAQ reagent [35]. This analysis yielded many proteins with significantly enriched binding to BAF-beads, relative to bead-only controls, expressed as enrichment ratios (Table 3).

**Table 1:** Summary of all non-redundant proteins identified in the 0.3 M and 1 M NaCl elutions of HeLa nuclear lysate proteins bound to BAF beads only.

GI	Name	Defline	Function	0.3 M no BAF	0.3 M BAF	1 M no BAF	1 M BAF
148529014	DDB1	Damage-specific DNA binding protein 1 (127 kD)	DNA repair (NER), ubiquitylation, transcription	0	9 (100)	0	0
4557515	DDB2	Damage-specific DNA binding protein 2 (48 kD)	DNA repair (NER), transcription	0	9 (100)	0	0
24047226	Mi2-b/CHD4	Chromodomain helicase DNA binding protein 4 (220 kD)	Chromatin remodeling, gene expression, rRNA trasncription	0	7 (100)	0	0
1016275	RBBP4/RbAp48	Retinoblastoma-binding protein mRbAp48 (52 kD)	Histone chaperone, chrom. remodeling, transcription, Ras signal., cytosk. org.	0	6 (100)	0	2 (99.8)
108860677	MDC1	Mediator of DNA damage checkpoint 1 (235 kD)	DNA repair, cell cycle checkpoints	0	5 (100)	0	0
109067205	HP1 gamma/CBX3	Pred: similar to chromobox homolog 3 (49 kD)	Chromatin binding, gene silencing	0	4 (100)	0	0
109095922	RECQL/RecQ1	Pred: similar to RecQ protein-like isoform 1 (88 kD)	DNA helicase, DNA repair	0	3 (99.9)	0	0
109107743	NUMA1	Pred: nuclear mitotic apparatus protein 1 isoform 3 (202 kD)	Mitotic spindle assembly	0	3 (99.9)	0	0
14211889	DPY-30 like	Dpy-30-like protein (11 kD)	Chromatin remodeling, gene expression, dosage compensation	0	3 (100)	0	2 (99.8)
109016427	GATA contain 2B	Pred: similar to GATA zinc finger domain containing 2B isoform 2 (65 kD)	Unknown	0	3 (100)	0	0
12052742	ACTL6A/BAF53a	Hypothetical protein (actin-like 6A/BAF53a)(47 kD)	Chromatin remodeling, transcription	0	3 (100)	0	0
1667394	HDAC1 or HDAC2	Transcriptional regulator homolog RPD3 (55 kD)	Chromatin remodeling, transcription	0	3 (100)	0	0
109081748	PKM	Pred: Pyruvate kinase 3 isoform 9 (65 kD)	Enzyme, phosphotyrosine binding protein	0	3 (100)	0	0
109132798	Filamin alpha	Pred: filamin 1 (actin-binding protein-280) isoform 4 (281 kD)	Actin cytoskeleton, signaling, trasncription	0	3 (100)	0	0
27529720	KIAA0670/Acinus	ACIN1 (apoptotic chromatin condensation inducer 1) homolog (146 kD)	DNA binding, apoptotic chromatin condensation	0	2 (99.7)	0	0
4885105	CAF-I p60	Chromatin assembly factor 1 subunit B (p60 subunit)(61 kD)	Chromatin structure and remodeling, transcription, DNA repair	0	2 (99.4)	0	0
109488886	HP1 beta/CBX1	Pred: similar to Chromobox protein homolog 1 (HP1 beta)(82 kD)	Chromatin binding, gene silencing	0	2 (99.2)	0	0
109105767	MTA2	Pred: similar to Metastasis-associated protein 2 (75 kD)	Chromatin remodeling, transcription	0	2 (99.1)	0	0
109082735	hnRNP C	Pred: Heterogeneous nuclear ribonucleoprotein C (C1/C2)(34 kD)	RNA binding	0	2 (98.4)	0	0
109130029	RBBP7/RbAp46	Pred: retinoblastoma binding protein 7 isoform 2, mRbAp46 (52 kD)	Histone chaperone, chromatin remodeling, transcription, Ras signaling	0	2 (97.7)	0	0
109097106	SMARCC2/BAF170	Pred: SWI/SNF-related matrix-assoc'd actin-dep.regulator of chrom. C2 (114 kD)	Chromatin structure and remodeling, transcription	0	2 (92)	0	0
28329430	Aprataxin/APTX	Aprataxin isoform b	DNA repair	0	1 (89.7)	0	0
15079335	SNRPD3/SMD3	Small nuclear ribonucleoprotein D3 (14 kD)	RNA binding, splicing activator	0	1 (89.7)	0	0
109508190	LOC690956	Pred: hypothetical protein	Unknown	0	1 (89.7)	0	0
178152	PARP1	Poly(ADP-ribose) polymerase (64 kD)	Chromatin structure and remodeling, transcription, DNA repair	0	1 (89.7)	0	0
109001148	HDAC1	Pred: similar to histone deacetylase 1 (54 kD)	Chromatin remodeling, transcription	0	1 (89.7)	0	0
109116261	U5-116KD/EFTUD2	Pred: U5 snRNP-specific protein (116 kD)	RNA binding, splicing activator	0	1 (89.7)	0	0
109042322	SFRS10	Pred: similar to Splicing factor arg/ser rich 10 (32 kD)	RNA binding, splicing activator	0	1 (89.7)	0	0
73951860	Limbin/EVC2	Pred: similar to Limbin (144 kD)	Unknown, related to skeletal development	0	1 (89.7)	0	0
109070808	HMGA1	Pred: similar to High mobility group AT-hook 1 isoform 2 (20 kD)	Chromatin structure, transcription, viral integration (HIV-1)	0	1 (89.7)	0	0
18644883	ATP5J	ATP synthase F0 complex subunit F6, H+ transporting (13 kD)	ATP synthase structure, H+ transport	0	1 (89.7)	0	0
27436951	Lamin B2	Lamin B2 (68 kD)	Nuclear architecture and function, signaling, gene expression	0	1 (89.7)	0	0
1711406	SMARCA2/BAF190	SWI/SNF-related matrix-assoc'd actin-dep.regulator of chrom. A2 (SNF2L2;181 kD)	Chromatin structure and remodeling, transcription	0	1 (89.7)	0	0
293686	Keratin 6A	Epidermal keratin subunit II (59 kD)	Structural	0	1 (89.7)	0	0
109504651	XP_573984	Pred: hypothetical protein (31 kD)	Unknown	0	1 (89.7)	0	0
156142197	G9a	Euchromatic histone-lysine N-methyltransferase 2 isoform a (EHMT2)(132 kD)	Chromatin remodeling, transcription	0	1 (89.7)	0	0
109076878	p15/SUB1/PC4	Pred: similar to Activated RNAP II transcriptional coactivator p15 (19 kD)	Chromatin remodeling, transcription (e.g., p53 gene), DNA repair	0	1 (89.7)	0	0
109128201	CD2BP2	Pred: CD2 antigen (cytoplasmic tail) binding protein 2 isoform 1 (37 kD)	Splicing, signaling	0	1 (89.7)	0	0
1083466	Requiem	Probable transcription factor requiem (42 kD)	Transcription factor, apoptosis	0	1 (89.7)	0	0
74136411	PAEP	Glycodelin, progesterone associated endometrial protein (21 kD)	Secreted protein	0	1 (89.7)	0	0
88953904	BAF/BANF1	Pred: similar to barrier-to-autointegration factor (10 kD)	Nuclear architect. & funct., chrom. org., gene express., viral integrat.	0	1 (89.7)	0	0
108935957	BCL7C	B-cell CLL/lymphoma 7 protein family member C (23 kD)	Unknown	0	1 (89.7)	0	0
109111495	LEDGF/p75	Pred: similar to PC4 and SFRS1 interacting protein 1 isoform 2	Transcriptional activator, mRNA splicing, viral integration (HIV-1)	0	1 (89.7)	0	0
124339826	Hsp70/HSPA1B	Heat shock 70 kDa protein 1B (70 kD)	Chaperone, stress response	0	1 (89.7)	0	0
115495195	Tektin 1	Tektin 1 (49 kD)	Structural, filament-forming protein localized in cilia and flagella	0	1 (89.7)	0	0
28279079	ZNF428	Zinc finger protein 428	DNA binding	0	1 (89.7)	0	0
1942991	Cyt C oxidase	Cytochrome C oxidase, Chain F (11 kD)	Electron transfer, metabolism	0	1 (89.7)	0	0
74220835	Lamin A/C	Unnamed protein product; lamin A/C (65 kD)	Nuclear architecture and function, signaling, gene expression	0	1 (89.7)	0	0
109074343	hnRNP D0/AUF1	Pred: similar to Heterogeneous nuclear ribonucleoprotein D0 (31 kD)	RNA binding, mRNA degradation	0	1 (89.7)	0	0
10181166	SMARCE1/BAF57	SWI/SNF related, matrix assoc'd, actin dep. regulator of chromatin E1 (47 kD)	Chromatin structure and remodeling, transcription	0	1 (89.7)	0	0
74207672	H2A	Unnamed protein product; putative H2A protein (14 kD)	Nucleosomes, chromatin organization and function	0	1 (89.7)	0	0
1195531	Keratin 16	Type I keratin 16	Structural	0	0	0	6 (100)
109096781	Keratin 6A	Pred: keratin 6A isoform 18	Structural	0	0	0	4 (100)
1722884	XPC/XPCC	DNA-repair protein, Xeroderma pigmentosum group C (106 kD)	DNA repair (NER)	0	0	0	3 (100)
109096823	Keratin 1	Pred: similar to keratin 1 isoform 7	Structural	0	0	0	2 (99.8)
1147813	Desmoplakin I	Desmoplakin I (DSP)	Cell-cell comunication (desmosomes), structural	0	0	0	2 (96.6)

Non-redundant protein hits for H_6_BAF-conjugated versus empty-control Ni-beads, eluted sequentially with 0.3 and 1 M NaCl, and identified with at least 89% confidence by Scaffold software. Columns labeled 0.3 M no BAF, 0.3 M BAF, 1 M no BAF and 1 M BAF indicate the number of unique peptides identified per elution, with the % confidence per each protein identified in brackets ( ). Abbreviations: GI, NCBI protein accession number; Defline, definition line/expanded description, plus predicted molecular weight by scaffold software in brackets ( ).

**Table 2:** Summary of all non-redundant proteins identified in the 0.3 M and 1 M NaCl elutions of HeLa nuclear lysate proteins bound to BAF and control beads.

GI	Name	Defline	Function	0.3 M no BAF	0.3 M BAF	1 M no BAF	1 M BAF
156523968	PARP1	Poly(ADP-ribose) polymerase family, member 1 (113 kD)	Chromatin structure and remodeling, transcription, DNA repair	0	31 (100)	1 (91.8)	11 (100)
27436946	Lamin A/C	Lamin A/C isoform 1, (70 kD)	Nuclear architecture and function, signaling, gene expression	24 (100)	25 (100)	5 (100)	12 (100)
109101818	Nucleolin	Pred: similar to nucleolin (101 kD)	Ribosome biogenesis, cell growth and proliferation, chromatin org.	6 (100)	10 (100)	5 (100)	2 (99)
11935049	Keratin 1	Keratin 1 (66 kD)	Structural	17 (100)	9 (100)	6 (100)	16 (100)
5031877	Lamin B1	Lamin B1 (66 kD)	Nuclear architecture and function, signaling, gene expression	1 (90.8)	8 (100)	0	2 (97.9)
10120888	BAF/BANF1	Breakpoint cluster region protein 1; barrier to autointegration factor 1 (10 kD)	Nuclear architect. and function, chrom. org., gene expression, viral integrat.	3 (100)	7 (100)	3 (100)	5 (100)
55956899	Keratin 9	Keratin 9 (62 kD)	Structural	3 (100)	6 (100)	0	8 (100)
109096963	HP1 alpha/CBX5	Pred: Chromobox protein homolog 5 (HP1alpha)(32 kD)	Chromatin binding, gene silencing	1 (90.8)	6 (100)	0	0
21961605	Keratin 10	Keratin 10 (59 kD)	Structural	8 (100)	5 (100)	5 (100)	7 (100)
109112233	Hsp70	Pred: heat shock 70 kDa protein 5 (glucose-regulated protein) isoform 1 (78 kD)	Chaperone, stress response	2 (99.8)	5 (100)	1 (91.8)	0
74003971	H2A	Pred: similar to histone 1, H2ai isoform 1 (29 kD)	Nucleosomes, chromatin organization and function	7 (100)	4 (100)	6 (100)	5 (100)
20336746	macro H2A	H2A histone family, member Y isoform 1 (39 kD)	Nucleosomes, chromatin organization and function (X inactivation)	6 (100)	4 (100)	4 (100)	5 (100)
109504921	H4	Pred: similar to germinal histone H4 (11 kD)	Nucleosomes, chromatin organization and function	6 (100)	4 (100)	0	0
109098344	TMPO/LAP2	Pred: Thymopoietin isoform 2 (LAP2)(75 kD)	Nuclear architecture and function, signaling	0	4 (100)	2 (99.8)	4 (100)
10835063	Nucleophosmin	Nucleophosmin 1 isoform 1 (33 kD)	Ribosome biogenesis, cell growth and proliferation, chromatin org.	5 (100)	3 (99.9)	4 (100)	5 (100)
109492380	Actin	Pred: similar to Actin, cytoplasmic 2 (Gamma-actin)(59 kD)	Structural, chromatin remodeling, transcription	5 (100)	3 (99.9)	5 (100)	3 (100)
47132620	Keratin 2	Keratin 2 (65 kD)	Structural	4 (100)	3 (99.9)	1 (91.8)	0
12653493	BASP1/NAP22	Brain abundant, membrane attached signal protein 1 (23 kD)	Nerve cell morphology, gene expression	2 (99.4)	3 (99.9)	2 (99.5)	2 (99.4)
109066680	H2A	Pred: similar to H2A histone family, member V isoform 1 (33 kD)	Nucleosomes, chromatin organization and function	4 (100)	3 (100)	3 (99.8)	3 (99.9)
109464859	Hsp70	Pred: similar to heat shock protein 8 (70 kD)	Chaperone, stress response	4 (100)	3 (100)	0	0
109071712	eEF1A 1	Pred: similar to eukaryotic translation elongation factor 1 alpha 1 isoform 4 (48 kD)	Protein synthesis	1 (90.8)	3 (100)	0	0
121903	H1.1 or H1.2	Histone H1.1, H1.2 (10 kD)	Nucleosomes, chromatin organization and function	2 (99.8)	2 (99.8)	5 (100)	0
225632	Casein	Casein alphaS1 (24 kD)	Secreted protein	1 (90.8)	2 (99.8)	0	0
109003367	EFIA/NSEP1/YB-1	Pred: similar to nuclease sensitive element binding protein 1 (49 kD)	DNA binding, gene expression	2 (97.7)	2 (99.1)	1 (91.8)	0
109114623	ASF/SF2/SFRS1	Pred: similar to splicing factor, arginine/serine-rich 1 (ASF/SF2)(45 kD)	RNA binding and splicing, genome stability, cell viability, viral splicing (HIV-1)	5 (100)	2 (98.1)	2 (99.8)	0
109070064	H1.5	Pred: similar to Histone H1.5 (H1a)(23 kD)	Nucleosomes, chromatin organization and function	3 (100)	2 (94.3)	2 (99.8)	2 (98.4)
157823451	RBMX/Rbmxrt	RNA binding motif protein, X chromosome retrogene-like (42 kD)	RNA binding, splicing activator	4 (100)	1 (89.7)	0	0
109121665	Myosin/MRCL2	Pred: similar to Myosin regulatory light chain 2, nonsarcomeric (20 kD)	Actin binding, stress fiber organization	3 (99.8)	1 (89.7)	2 (99.8)	0
109067280	Myosin/MLC6	Pred: similar to Myosin light polypeptide 6 (17 kD)	Motor protein	2 (99.6)	1 (89.7)	2 (99.2)	0
12803709	Keratin 14	Keratin 14 (epidermolysis bullosa simplex, Dowling-Meara, Koebner)(52 kD)	Structural	2 (98.9)	1 (89.7)	0	13 (100)
16418453	Pannexin 3	Pannexin 3, putative gap junction protein pannexin 3 (45 kD)	Cell-cell comunication (cell type-specific gap junctions)	1 (90.8)	1 (89.7)	1 (91.8)	0
126165296	H2B	Histone cluster 1, H2bn (14 kD)	Nucleosomes, chromatin organization and function	1 (90.8)	1 (89.7)	1 (91.8)	0
10645195	H2A	Histone cluster 1, H2ae (14 kD)	Nucleosomes, chromatin organization and function	1 (90.8)	1 (89.7)	1 (91.8)	0
12653649	RPL14	Ribosomal protein L14 (24 kD)	Ribosome biogenesis	1 (90.8)	1 (89.7)	1 (91.8)	0
41946811	SNRPF/snRNP F	Snrpf protein, small nuclear ribonucleoprotein polypeptide F (12 kD)	snRNPs biogenesis, RNA splicing	1 (90.8)	1 (89.7)	0	0
57110429	H1.5	Pred: similar to Histone H1.5 (H1a)(22 kD)	Nucleosomes, chromatin organization and function	1 (90.8)	1 (89.7)	0	0
6005757	SPT16/FACTp140	Chromatin-specific transcription elongation factor large subunit (120 kD)	Replication, transcription, nucleosome disassembly, H2AX exchange factor	3 (99.9)	0	1 (91.8)	6 (100)
109096789	Keratin 5	Pred: keratin 5 isoform 15 (63 kD)	Structural	3 (99.8)	0	1 (91.8)	11 (100)
21754583	Keratin	Unnamed protein product; similar to keratin	Structural	2 (99.8)	0	0	3 (100)
109106294	SSRP1/FACTp80	Pred: similar to structure specific recognition protein 1 (81 kD)	Replication, transcription, nucleosome disassembly, H2AX exchange factor	2 (98)	0	2 (99.8)	3 (100)

Non-redundant protein hits for H_6_BAF-conjugated versus empty-control Ni-beads, eluted sequentially with 0.3 and 1 M NaCl, and identified with at least 89% confidence by Scaffold software. Columns labeled 0.3 M no BAF, 0.3 M BAF, 1 M no BAF and 1 M BAF indicate the number of unique peptides identified per elution, with the % confidence per each protein identified in brackets ( ). Abbreviations: GI, NCBI protein accession number; Defline, definition line/expanded description, plus predicted molecular weight by scaffold software in brackets ( ).

**Table 3:** iTRAQ quantification of bound proteins eluted by 0.3 M or 1.0 M NaCl from BAF beads, relative to bead-only controls.

Score	% Cov	GI	Name	Description	0.3 M/control	1M/0.3 M
59.9	56.2	125962	LMNA_HUMAN	Lamin-A/C (70 kDa lamin)	11.1	0.3
18.6	44.2	136429	TRYP_PIG	Trypsin precursor	1.1	1.2
14.9	94.4	71152308	BAF_PONPY	Barrier-to-autointegration factor	19.1	1.5
10.9	41.2	114762	NPM_HUMAN	Nucleophosmin (NPM)	15.9	2.6
7.2	73.8	81884120	H2B1H_MOUSE	Histone H2B	10.8	3.0
6.9	19.9	1709851	SFPQ_HUMAN	Splicing factor, proline- and glutamine-rich	3.5	0.4
4.5	23.7	1168325	ACT5_BACDO	Actin	3.7	0.8
4.4	21.1	46396655	SET_MOUSE	SET (Protein Phosphatase 2A inhibitor, I2PP2A)	20.6	0.8
4.0	10.7	38257560	FLNA_MOUSE	Filamin-A	–	–
4.0	22.1	67460593	NONO_RAT	NonO protein	3.1	0.4
3.7	34.0	51315709	H4_MYTCH	Histone H4	10.7	0.9
3.4	35.3	73919839	H33_VITVI	Histone H3.3	8.8	1.4
3.3	13.4	133274	HNRPL_HUMAN	Heterogeneous nuclear ribonucleoprotein L (hnRNP L)	1.8	0.6
3.3	40.4	75277395	H2AV1_ARATH	Histone H2A	9.5	4.0
2.9	32.4	417101	H12_HUMAN	Histone H1.2	7.6	0.9
2.5	11.7	1730009	TPR_HUMAN	Nucleoprotein TPR	3.6	0.5
2.1	17.1	26390818	AN32B_HUMAN	Acidic leucine-rich nuclear phosphoprotein (PHAPI2)	6.7	0.6
2.1	9.9	61214649	PP1RA_PIG	Serine/threonine-protein phosphatase 1 (FB19)	3.4	0.6
2.0	8.6	1708162	HD_RAT	Huntingtin (Huntington disease protein)	15.0	1.5
2.0	8.4	40739534	KLPA_EMENI	Kinesin-like protein klpA	–	–
2.0	17.7	90110023	H2AY_HUMAN	Core histone macro-H2A (inactive X chromosome)	13.5	2.0
2.0	9.2	82236756	RBP4B_XENLA	Histone-binding protein RBBP4/7	4.5	0.9
2.0	12.8	71162379	SLYD_SHIFL	FKBP-type peptidyl-prolyl cis-trans isomerase slyD	49.6	0.3
1.7	16.1	547754	K22E_HUMAN	Keratin	3.7	0.8
1.7	12.6	12229941	PSA5_SCHPO	Probable proteasome subunit alpha type 5	–	–
1.4	8.7	6685905	RPOC2_ARATH	DNA-directed RNA polymerase beta chain	43.4	1.1
1.2	11.6	122066350	TCOF_HUMAN	Treacle protein (Treacher Collins syndrome protein)	14.5	3.2

Abbreviations: Score, protein pilot score (2 pts per unique 99% confidence peptide, 1 pt per unique 95% confidence peptide); % Cov, percent peptide coverage of identified protein; GI, NCBI protein accession number; 0.3 M, 0.3 M NaCl eluate; 1 M, 1.0 M NaCl eluate.

The iTRAQ analysis (Table 3) yielded several abundant proteins known to bind BAF directly, including lamin A [12], core histones H3 and H4 [28], plus one protein, histone H1.2, known to associate with BAF indirectly [28]. All detected proteins were present in both the 0.3 and 1 M BAF-bead eluates (Table 3; 1 M/0.3 M column). Several proteins including H2A, H2B and Treacle were enriched 3–4 fold in the 1 M eluate, compared to the 0.3 M eluate (Table 3; 1 M/0.3 M column), suggesting relatively high affinity either for BAF or a co-purified partner (e.g., lamin A or histone H3 [12], [28]). The iTRAQ results for the 0.3 M eluates showed that lamin A/C and all four core histones were enriched on BAF-beads (over control beads) by ∼11-fold and 8.8-to-10.8-fold, respectively (Table 3; 0.3 M/control column). Similar enrichments for individual core histones implied similar stoichiometry and suggested they bound BAF as nucleosomes. This was consistent with evidence that BAF binds core histone H3 *in vitro* and *in vivo* [28] and associates with nucleosomes *in vivo* (Montes de Oca, Andreassen & Wilson, in preparation). Alternatively, core histones might co-enrich on BAF-beads by binding other abundantly recovered proteins including lamins (via H2A/H2B; [36]) or PARP1 [37]. Histone macro-H2A, which concentrates on the inactive X-chromosome [38], was enriched 13.5-fold (Table 3, 0.3/control column). Compared to controls, the iTRAQ analysis (Table 3) also revealed significant enrichment for nucleophosmin (∼16-fold), SET/I2PP2A (∼21-fold), transcription factor NonO (∼3-fold), Ser/Thr-protein phosphatase 1 (FB19; ∼3-fold), Huntingtin (15-fold), RBBP4 and/or RBBP7 (∼4-fold; see also Table 1), FKBP-type peptidyl-prolyl cis-trans isomerase slyD (∼50-fold), DNA-directed RNA polymerase beta (∼43-fold) and Treacle (Treacher-Collins Syndrome protein; ∼14-fold).

Additional candidates were independently identified by LC-MS/MS (Tables 1–2) including three transcription factors (Requiem, p15/SUB1/PC4 and LEDGF/p75 [Table 1]) and a remarkable number of proteins involved in gene regulation or genome integrity including PARP1 (Tables 1–2), DDB1 and DDB2 (Table 1) and Xeroderma pigmentosum complementation group C protein (XPC/XPCC; Table 1). Also recovered were Mediator of DNA damage Checkpoint 1 protein (MDC1; which associates with phosphorylated H2AX [γH2AX] and Mre11 at sites of DNA damage [39]; Table 1), and five components of the nucleosome remodeling and histone deacetylase (NuRD) complex: unique components MTA2 and Mi2β/CHD4 [40], [41] (Table 1) plus HDAC1 or HDAC2 (unique peptides not recovered; Table 1), and RBBP4 and RBBP7 (Tables 1, 3). Also identified were DPY-30-like protein (a component of Set1 histone methyltransferase complexes [42]; Table 1), G9a (an H3-K9 histone methyltransferase [43], [44]; Table 1) and three isoforms of heterochromatin protein 1 (HP1α, HP1β, HP1γ; Tables 1–2), which specifically recognize and bind K9-methylated H3 [45], [46].

### Independent validation of selected candidates

**Figure 2:**
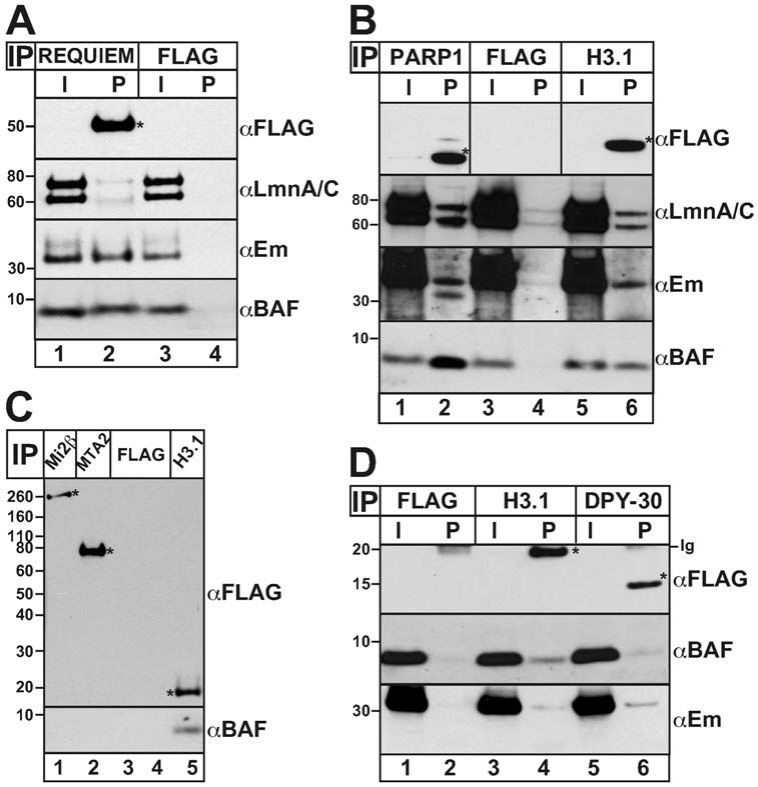
BAF associates with Requiem and PARP1 *in vivo*. Western blot analysis of immunoprecipitates from HeLa cells that transiently expressed either FLAG-Requiem (A), FLAG-PARP1 (B), FLAG-Mi2β or FLAG-MTA2 (C), FLAG-DPY-30-like (D), FLAG-H3.1 (positive control) or FLAG vector alone (negative control). Samples were precipitated with Flag-M2 conjugated beads and eluted with 2X-SB except in (B) where bound proteins were eluted using Flag-peptide (see Methods). Input (I, 1%) and pellet (P, 50%) fractions were western blotted using antibodies specific for FLAG (αFLAG), BAF (αBAF), emerin (αEm) or lamins A and C (αLmn A/C) which migrate at 70 and 60 kD, respectively. Asterisks (*) indicate each transiently expressed protein. Representative results are shown for Requiem (n>4), PARP1 (n>4), Mi2β and MTA2 (pellets only; n = 3) and DPY-30-like (n>4).

**Figure 3:**
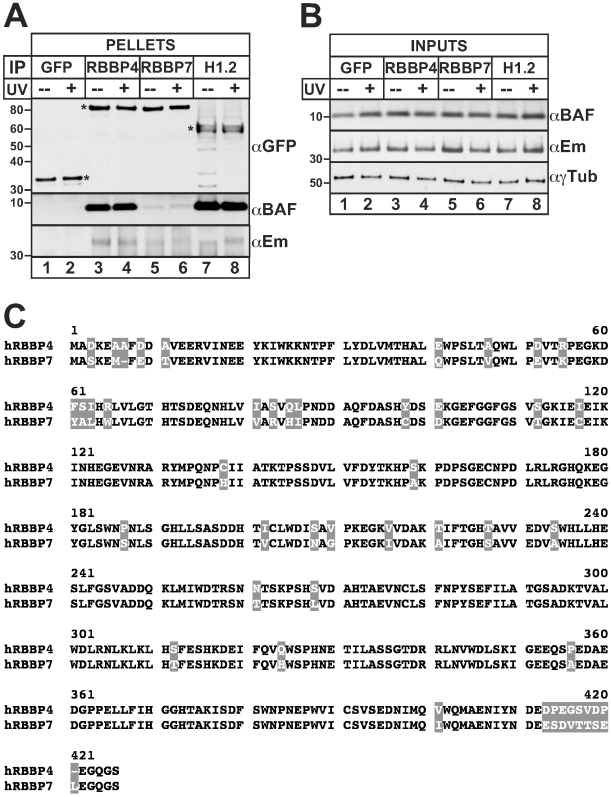
BAF associates efficiently with RBBP4, and weakly with RBBP7, in HeLa cells. Western blot analysis of HeLa cells that transiently expressed GFP-RBBP4, GFP-RBBP7, GFP-H1.2 (positive control, BAF-associated *in vivo*) or GFP (negative control) for 24 hrs. Cells were then either left untreated (−) or UV-irradiated (+), and allowed to recover for 1 hr in fresh media prior to harvest. Western blots of (A) GFP-immunoprecipitates (50%), or (B) input whole cell lysates (0.5%), were probed with antibodies specific for GFP (αGFP), BAF (αBAF), emerin (αEm) or γTubulin (αγTub; loading control). Asterisks (*) indicate each transiently expressed protein. Representative results are shown (n = 3). (C) Aligned amino acid sequences of human RBBP4 and RBBP7, with non-identical residues shaded in gray (89.4% identity).

**Figure 4:**
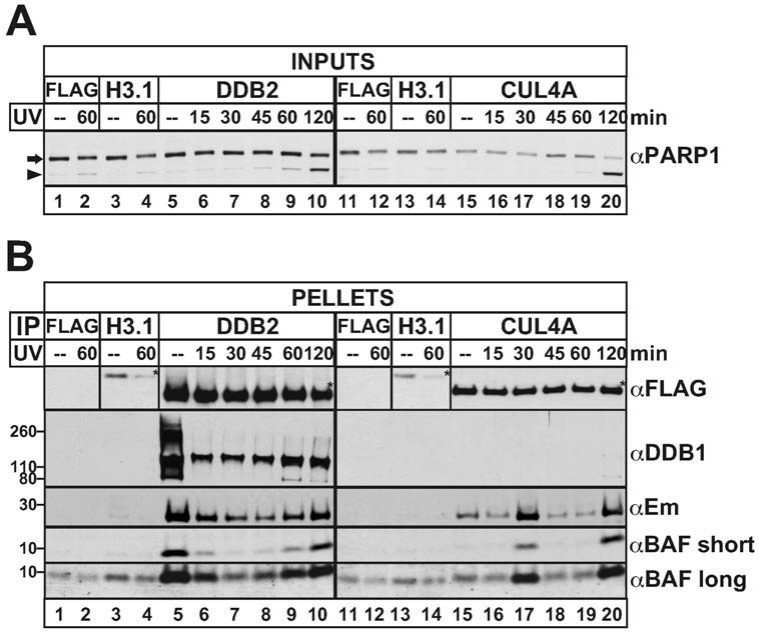
Dynamic associations of endogenous BAF and emerin with DDB2 and CUL4A in UV-treated cells. Western blot analysis of HeLa cells that transiently expressed FLAG-CUL4A, FLAG-DDB2, FLAG-H3.1 (positive control) or FLAG alone (negative control) for 24 hrs. Cells were either left untreated and harvested at the 1 hr timepoint (−), or UV-irradiated and allowed to recover in fresh media for the indicated time prior to harvest. Western blots of (A) input whole cell lysates (2% loaded) or (B) the corresponding FLAG-immunoprecipitates (50% of controls loaded; other samples 100% loaded), probed with antibodies specific for FLAG (αFLAG), BAF (αBAF, short and long exposures), emerin (αEm), DDB1 (αDDB1; positive control for DDB2 interaction) or PARP1 (αPARP1). The PARP1 antibody recognizes both full-length PARP1 (arrow) and a caspase-cleaved PARP1 fragment (arrowhead), indicative of apoptosis. Asterisks (*) indicate each transiently expressed FLAG-tagged protein. Results shown are representative of at least three independent experiments.

To evaluate the physiological relevance of this proteome, we chose 11 candidates to test independently for potential association with BAF *in vivo*. Antibodies for many candidates were unavailable or unsuitable for immunoprecipitation, so we used an epitope-tag strategy. Each candidate was transiently expressed as a FLAG- or GFP-tagged protein in HeLa cells, then immunoprecipitated from whole cell lysates using antibodies against the epitope tag, resolved by SDS-PAGE and western blotted using antibodies specific for either the tag, endogenous BAF or other endogenous proteins (Figures 2, 3, 4). Compared to the positive and negative controls (FLAG-H3.1 and FLAG vector, respectively), BAF co-precipitated reproducibly with G9a and SET/I2PP2A (Montes de Oca, Andreassen & Wilson, in preparation) and five other candidates including Requiem (Figure 2A) and PARP1 (Figure 2B), as detailed below. By contrast, four other candidates associated with BAF undetectably or weakly, under the same immunoprecipitation conditions: Mi2β and MTA2 (Figure 2C, lanes 1 and 2 *vs* 3 and 4; n = 3), HDAC1 (data not shown) and DPY-30-like (Figure 2D, lane 6 *vs* 2, αBAF; n>4).

### BAF associates with Requiem *in vivo*

Requiem, with a *Kruppel*-type zinc finger and four atypical zinc-fingers, is a pro-apoptotic transcription factor in myeloid cells homologous to the mouse Ubi-d4 protein [47], [48]. FLAG-Requiem reproducibly (n>4) and specifically co-immunoprecipitated both endogenous BAF and endogenous emerin from cells (Figure 2A, lane 2, αBAF and αEm). These results independently validated Requiem as BAF-associated *in vivo*, and further suggested potential ternary interactions with emerin. Whether BAF or emerin (or both) bind Requiem directly was not tested.

### BAF associates with PARP1 in HeLa and HEK293 cells

PARP1, represented by at least 31 distinct peptides, was the most significant protein identified by LC-MS/MS (Tables 1–2). PARP1 ADP-ribosylates many substrates including histones, regulates chromatin structure and has well-characterized roles in DNA damage responses [49]. We therefore independently tested a potential association between FLAG-PARP1 and endogenous BAF in HeLa cells. Controls showed little or no background pelleting of endogenous BAF with FLAG vector alone (Figure 2B, lane 4, αBAF), and positive co-precipitation with FLAG-H3.1 (Figure 2B, lane 6, αBAF), as expected. Endogenous BAF co-precipitated reproducibly and at high levels with FLAG-PARP1 under both low stringency (150 mM NaCl, 0.15% TX-100; data not shown), and high-stringency conditions (300 mM NaCl, 0.3% TX-100; Figure 2B, lane 2, αBAF; n>4). A reciprocal experiment using a stable, tetracycline-inducible FLAG-BAF overexpressing cell line (HEK293, human embryonic kidney; [50]) gave similar results: endogenous PARP1 co-immunoprecipitated with FLAG-BAF (data not shown). Further Western blotting of the FLAG-PARP1 immunoprecipitates confirmed the presence of lamins A and C (Figure 2B, lane 2, αLmnA/C; n>4) under high stringency conditions, as expected [51], [52]; and also revealed low levels of the nuclear membrane protein emerin (Figure 2B, lane 2, αEm; n>4), with higher signals under low stringency conditions (not shown). These results suggest BAF associates with PARP1 in two independent cell lines: HeLa and HEK293.

### Selective association of BAF with RBBP4 in HeLa cells

RBBP4 (also known as Chromatin Assembly Factor-1 [CAF-1] p48 subunit or RbAp48) and RBBP7 (RbAp46) are homologous histone chaperones encoded by different genes. Both were specifically identified by LC-MS/MS (Table 1) and at least one was identified by iTRAQ (Table 3). RBBP4 and RBBP7 are WD-repeat proteins that contribute, either together or separately, to several chromatin-modifying complexes including the NuRD complex [40], [41] and the CAF-1 complex, required for chromatin assembly after DNA replication and repair [53], [54]. We tested for potential interaction between endogenous BAF and either GFP-RBBP4 or GFP-RBBP7 in HeLa cells under normal conditions, or 1 hr after UV-irradiation, since RBBP4 and the CAF-1 complex mediate histone deposition during UV-damage repair. Controls confirmed little association of BAF with GFP alone (Figure 3A, lanes 1 and 2, αBAF) and positive association with GFP-H1.2 (Figure 3A, lanes 7 and 8, αBAF), as expected [28]. Endogenous BAF co-precipitated robustly with GFP-RBBP4 in both undamaged and UV-treated cells (Figure 3A, lanes 3 and 4, respectively, αBAF; n = 3). By contrast, near background levels of BAF co-precipitated with GFP-RBBP7 (Figure 3A, lanes 5 and 6, αBAF; n = 3). Endogenous emerin co-precipitated very weakly with both GFP-RBBP4 and GFP-RBBP7 (Figure 3A, lanes 3–6, αEm; n = 3). Control Western blots of whole cell lysates verified that UV-irradiation did not significantly alter the levels of endogenous BAF or emerin proteins, relative to loading control γ-tubulin (Figure 3B, inputs; n = 3). BAF interaction with RBBP4 was further confirmed in reciprocal studies of stable HEK293 cells with tetracycline-inducible expression of FLAG-BAF (as above): FLAG-BAF co-immunoprecipitated endogenous RBBP4 (data not shown). Thus, despite 89.4% amino acid identity between RBBP4 and RBBP7 (Figure 3C) [55], BAF associated robustly and selectively with RBBP4 in both untreated and UV-treated cells.

### BAF and emerin interactions with DDB2 and CUL4 in UV-irradiated HeLa cells

Three proteomic candidates (DDB1, DDB2, XPC; Table 1) are involved in DNA damage repair. DDB1 and DDB2 are key components of the UV-DNA-damage-binding protein complex (UV-DDB) [56], which recognizes DNA-distorting lesions and mediates nucleotide-excision repair, in part by recruiting XPC-RAD23B heterodimers [57]. In addition, DDB1 and DDB2 also associate with Cullin 4 (CUL4)-containing E3 ubiquitin ligase complexes. One such complex, CUL4-DDB-ROC1, is recruited rapidly (5–10 min) by DDB1-DDB2 to sites of UV-induced DNA damage, where it ubiquitylates H3 and H4 causing nucleosome eviction and exposure of damaged DNA [58]. The CUL4-DDB-ROC1 complex then promotes repair by recruiting repair proteins XPC and its partner RAD23B to damage sites [59].

We found that endogenous DDB1 co-immunoprecipitated with FLAG-BAF in tetracycline-induced HEK293∶BAF cells (data not shown), independently validating its association with BAF *in vivo*. Given the above-described roles for DDB1-DDB2 and XPC-RAD23B, and recognizing that CUL4 was not identified in our (unirradiated) HeLa cell proteome, we hypothesized that BAF might be involved in cellular responses to UV-damage. To test this idea we expressed either FLAG-CUL4A, FLAG-DDB2, FLAG-H3.1 (positive control) or FLAG vector alone (negative control) transiently (∼24 hrs) in HeLa cells. Cells were then either treated with UV light (20 J/m^2^) or left untreated as controls, and allowed to recover for the indicated time. Whole cell lysates were then prepared and either Western blotted directly (Figure 4A, inputs) or after immunoprecipitation with FLAG-conjugated beads (Figure 4B, pellets). To assess whether cells were effectively damaged by UV-treatment, whole cell lysates were Western blotted with antibodies against endogenous PARP1 to assay production of its characteristic apoptosis-induced cleavage product [60]. PARP1 cleavage was weak or undetectable in untreated cells (Figure 4A, “—”; arrowhead), and detectable but faint 60 min after UV-treatment (Figure 4A, lanes 2, 4, 9, 12, 14, 19; arrowhead; n = 3). PARP1 cleavage was obvious 120 min after UV-treatment (Figure 4A, lanes 10 and 20; arrowhead), suggesting cells had entered apoptosis. FLAG-pulldowns from untreated cells or from each timepoint after UV-irradiation, were probed with antibodies specific for either FLAG, endogenous DDB1, endogenous emerin or endogenous BAF (Figure 4B). Endogenous DDB1 co-precipitated with FLAG-DDB2 at all timepoints (Figure 4B, lanes 5–10, αDDB1; n = 3) as expected, since these proteins form heterodimers [61]. Validating our proteomic results, endogenous BAF co-precipitated with FLAG-DDB2 (Figure 4B, lanes 5–10, αBAF; short and long exposures both shown; n = 3) above the FLAG vector control background (Figure 4B, lanes 1–2, αBAF; n = 3), in both UV-treated and untreated cells. Endogenous emerin also co-precipitated efficiently with FLAG-DDB2 (Figure 4B, lanes 5–10, αEm; n = 3) in both UV-damaged and undamaged cells. In addition, emerin also co-precipitated with FLAG-CUL4A in undamaged cells, and this association transiently decreased, then increased after UV-treatment (Figure 4B, lanes 15–20, αEm; n = 3). A similar trend was seen for the endogenous BAF association with FLAG-CUL4A (Figure 4B, lanes 15–20, αBAF; n = 3). Despite experiment-to-experiment variation in the exact timing of highest association between BAF or emerin and FLAG-CUL4A, endogenous BAF and emerin consistently “co-peaked” in their interaction with FLAG-CUL4A after UV-treatment. These results validated CUL4A, DDB2 and DDB1 as partners for BAF *in vivo*, and suggested their association with BAF is differentially regulated in response to UV-damage. Furthermore, we discovered robust and potentially UV-regulated associations of the nuclear membrane protein emerin with both DDB2 and CUL4A. These results suggest novel roles for both BAF and emerin in genome integrity.

## Discussion

Many pathways including transcription, replication, DNA repair, signaling and chromosome tethering require an intact nuclear lamina network [2], [5]–[8]. The mechanisms of this dependence are major questions in biology with relevance to aging and a spectrum of human diseases. BAF is an essential component of the nuclear lamina network, interacting with both A- and B-type lamins as well as nuclear membrane proteins and chromatin [13]. Our use of two complementary proteomic methods (LC-MS/MS and iTRAQ), and comparisons to negative controls, yielded over 70 potential interactors. Many candidates are likely to bind lamins directly, rather than BAF, and comprise a “lamin proteome” within the BAF proteome. BAF has nanomolar to low micromolar affinity for key partners (histones, LEM-domain proteins, lamins) [12], [28], [62] and can stabilize “ternary” interactions between lamin A and emerin [63]. BAF-mediated stabilization of ternary complexes might account for the quantitative enrichments of lamins and histones (and potentially other candidates, e.g., SET/I2PP2A, Huntingtin) detected by iTRAQ analysis. The iTRAQ quantification was important because many nuclear proteins including LAP2, lamins and histones have been reported to bind beads nonspecifically [64], and were also detected by LC-MS/MS in our control eluates (Table 2). Confidence that this proteome includes novel interactors for BAF was increased by independent validation: seven of 11 tested FLAG- or GFP-tagged candidates, including single-peptide “hits” Requiem (reported here) and G9a (Montes de Oca, Andreassen & Wilson, in preparation), co-precipitated endogenous BAF from HeLa cells. An important caveat is that these verifications were based on transient overexpression of epitope-tagged candidates, which might cause artifacts. It will be important in the future to verify the interaction between endogenous BAF and each endogenous partner.

### Other candidates

Other interesting candidates await investigation. For example the four non-skin keratins (keratins 5, 6A, 14, 16) [65] identified in the high-stringency (1 M salt) BAF-bead eluate (Tables 1–2) suggest that BAF, which can reside in the cytoplasm (where it mediates the innate immune response to poxvirus infection [50]), might interact with both nuclear and cytoplasmic intermediate filaments. Other untested candidates include Huntingtin and Treacle (Table 3), acinus (Table 1; involved in internucleosomal DNA cleavage during apoptosis [66]) and MDC1 (Table 1; binds repair proteins and mediates DNA-damage checkpoints during S-phase [67] and the G2/M transition [68]). Based on the extended S-phase observed in BAF-deficient cells [31] and unexplained positive roles for the LEM-domain protein LAP2 in DNA replication [69], we speculate BAF might facilitate DNA replication by influencing MDC1 or PARP1 (Tables 1–2; [70]), or by facilitating chromatin reorganization. Besides G9a (an H3-K9 methyltransferase), this proteome also included heterochromatin proteins HP1α, β and γ (Tables 1–2), which specifically bind G9a itself and K9-methylated H3 and mediate long-term silencing and heterochromatin formation [71]. We also specifically recovered four subunits of SWI/SNF complexes (Table 1): SMARCA2/BAF190 (also known as Brahma/BRM), and three Brahma-associated factors (regrettably, also known as BAFs): SMARCC2/BAF170, SMARCE1/BAF57 and the actin-related protein BAF53A/ACTL6A. SWI/SNF complexes are ATP-dependent chromatin remodeling complexes involved in gene expression, differentiation, proliferation and DNA repair [72]. We are unaware of any previous evidence that potentially links SWI/SNF complexes to lamins or BAF (Barrier-to-Autointegration Factor), and this will be important to test in future work.

### Four human transcription factors that potentially bind BAF

BAF can regulate tissue-specific gene expression in *C. elegans* by binding and occupying specific promoters [26]. In mammalian retinal cells, BAF is known to bind directly to Crx and related homeodomain transcription factors and represses Crx-dependent gene activation [32]. Whether BAF binds other transcription factors, or regulates specific genes in other human tissues, is largely unexplored. Our proteome identified four transcription factors (p15/SUB1/PC4, NonO, Requiem and LEDGF) with which BAF might associate in human cells. p15/SUB1/PC4 (Table 1; hereafter “PC4”) is an abundant nuclear protein that binds p53 and also transcriptionally co-activates p53 function [73]. The p53 protein, in turn, activates PC4 transcription as part of a feedback loop [74]. Interestingly, PC4 also binds histones, condenses chromatin [75] and helps repair damaged DNA [76], [77]. NonO (aka p54^nrb^) (Table 3), binds and helps SWI/SNF complexes activate transcription, and might be involved in splicing of the telomerase reverse transcriptase (*TERT*) gene [78]. Requiem (single-peptide “hit”; Table 1), validated in this study as BAF-associated *in vivo*, is a relatively uncharacterized transcription factor that regulates apoptosis in the myeloid lineage [47], [48]. LEDGF/p75 (single-peptide “hit”; Table 1) is a transcription factor that can bind human immunodeficiency virus type 1 (HIV-1) integrase directly, and contributes significantly to the efficiency and location of HIV-1 integration sites in human chromosomes [79]. BAF binds the HIV-1 matrix and Gag proteins directly, and is a component of HIV-1 virions [80]. BAF and emerin were both reported to contribute significantly to the efficiency of HIV-1 integration into primary human macrophages [33]. Two subsequent studies of emerin-null cells failed to support this role for emerin [34], [81], and one study reported that siRNA-mediated downregulation of BAF had only modest effects on HIV-1 infectivity in HeLa-P4 cells [34]. Further studies of macrophages and other primary cells are needed to test the proposed contribution of BAF to HIV-1 infection. Our results, which suggest a potential association between BAF and LEDGF, present a new way to study this question in HIV-1-infected primary cells.

### BAF association with PARP1

BAF associated robustly *in vivo* with our best proteomic “hit”, PARP1. PARP1 transfers ADP-ribose units to itself and other proteins in an NAD^+^-dependent manner. PARP1 can promote higher-order compaction of nucleosome arrays and repress transcription [37], [82], but can also activate transcription and has major roles in DNA repair [83]. Our BAF proteome also included RecQ protein-like (Table 1), a DNA helicase involved in DNA repair that can associate with PARP1 [84]. PARP1 was shown previously to interact with A-type lamins [51], [52]; our findings suggest these ternary complexes also include BAF, and a subset might also associate with emerin at the INM. How and when BAF associates with PARP1, and whether the BAF-PARP1 interaction includes RecQ, or requires lamins, are important new questions.

### Implications of BAF association with RBBP4

Our independent validation suggests BAF associates preferentially with RBBP4, not RBBP7, under similar immunoprecipitation conditions. RBBP4 and RBBP7 are 89.4% identical, and the majority of the amino acid changes are conservative substitutions (Figure 3C, grey boxes). Thus, BAF selectivity for RBBP4 over RBBP7 might be due to recognition of residues unique to RBBP4, or to an intermediary protein that discriminates RBBP4 from RBBP7. Understanding the basis for this preference might shed light on how DNA replication and DNA repair depend on the nuclear lamina network [7], [85], [86]. RBBP4 is a chaperone common to H3.1, H3.3 and CenH3 deposition complexes, which assemble H3 into nucleosomes both during and independently of DNA replication [87], [88]. BAF can bind H3.1 directly [28], and has intriguing behavior in primary cells: BAF localizes to both the nucleus and cytoplasm of interphase cells except during S-phase, when it becomes exclusively nuclear [31]. S-phase is prolonged four hours in HeLa cells downregulated for BAF, suggesting BAF somehow facilitates S-phase progression [31], through unknown mechanisms. RBBP4, as a component of the CAF-1 complex, associates indirectly with PCNA (a lamin A-binding protein [86]) to promote chromatin assembly on newly-replicated or newly-repaired DNA [89]. We propose BAF might dynamically stabilize chromatin complexes that include RBBP4 and PCNA by helping tether these complexes to lamins, analogous to the proposed BAF-mediated stabilization of emerin binding to lamin A [63]. Our proteome included another CAF-1 subunit, p60 (Table 1); whether BAF associates with the CAF-1 complex itself remains to be tested. We also recovered both SPT16/p140 (Table 2), the large subunit of the Facilitates Chromatin Transcription (FACT) chaperone complex, and its binding partner SSRP1/FACT p80 (Table 2) which are involved in DNA replication, transcription and repair [90]. RBBP4 (validated) and the FACT complex (not yet independently tested) each suggest mechanisms by which BAF might link chromatin assembly, replication, repair and other activities to the nuclear lamina network.

### Proposed novel roles for BAF and emerin in DNA damage response

Human cells that transiently express certain laminopathy-causing lamin A mutations, show either reduced formation of γH2AX foci after DNA damage [7] or higher proportions of γH2AX-positive cells in the absence of damage [91]. This suggests that DNA damage responses require an intact lamina network. Furthermore, nuclear alpha-II-spectrin, which binds and recruits specific repair proteins to sites of DNA damage [92], also co-precipitates with lamin A and emerin [93] and co-purifies with emerin and lamin A in multi-protein complexes isolated from HeLa cells [94]. Our new results independently implicate both BAF and emerin in cellular responses to DNA damage. In undamaged HeLa cells, both proteins associated with CUL4A and DDB2; and FLAG-BAF also associated with endogenous DDB1 in HEK293∶BAF cells (unpublished observations), implying some kind of ongoing molecular surveillance or regulation that involves the nuclear envelope (emerin) and BAF. Our results suggest these interactions are regulated differentially after UV-induced damage and also potentially by apoptotic signaling, since the associations also increased at the time of PARP1 cleavage (120 min). We propose that BAF and emerin have dynamic and potentially regulated roles after DNA damage. Whether BAF and emerin function independently or concertedly, and whether their roles include the actual repair of damaged DNA, are open questions.

Our major conclusion is that this BAF proteome, validated for seven of 11 tested candidates, is a rich source of candidate proteins that might reveal how DNA damage responses, genome replication, epigenetic control and chromatin organization might be supported by BAF, and might be dynamically coupled to the nuclear envelope and nuclear lamina networks.

## Materials and methods

### Cell culture, nuclear protein lysates and affinity purification using BAF-beads

HeLa cells were obtained from the American Type Culture Collection and cultured in Dulbecco's modified eagle's medium containing 10% fetal bovine serum. To prepare protein lysates, HeLa cells were resuspended in hypotonic buffer (10 mM HEPES pH 8, 10 mM KCl, 0.1 mM EDTA, 0.1 mM EGTA, 1 mM DTT, 0.5% NP-40), incubated on ice 10 min and then lysed by 8–10 passes through a 26G3/8 needle and syringe. Lysates were centrifuged (5000 rpm, 10 min, 4°C) to obtain the nuclear pellet, and nuclear proteins were solubilized by gentle rotation at 4°C in lysis buffer (20 mM HEPES pH 8, 150 mM NaCl, 0.1% Triton X-100, 0.2 mM EDTA, 0.2 mM EGTA, 1.5 mM MgCl_2_, 2 mM DTT, benzonase to digest DNA, and protease inhibitors) for 30 min; samples were then centrifuged (5,000 rpm, 5 min, 4°C) to remove insoluble material, and solubilized nuclear lysate proteins were either used fresh or stored at −80°C after being flash frozen with liquid nitrogen.

BAF-conjugated beads were made by incubating 30 µg purified isolated H_6_BAF dimers prepared by affinity purification and size exclusion chromatography as described [23], [28], with 30 µl Ni-NTA beads (Qiagen, Valencia, CA) overnight at 4°C, at a final protein concentration of 1 µg/μl. Control beads were incubated with lysis buffer only. Ni-alone or Ni-BAF beads were then each washed twice with lysis buffer and incubated with 500 µg pre-cleared nuclear lysate proteins, rotating, for 3 hrs at 4°C. Unbound material was removed by four washes with lysis buffer lacking detergent. Bound proteins were eluted sequentially, first with lysis buffer containing 0.3 M NaCl (no detergent), then with lysis buffer containing 1 M NaCl (no detergent), both compatible with in-solution trypsin digestion for LC-MS/MS, and finally with 0.5% SDS. The remaining beads were resuspended in 2X-SDS sample buffer (2X-SB). About 10% of each eluate was resolved by SDS-PAGE and silver stained for qualitative analysis. Two pairs of samples were analyzed in solution by LC-MS/MS: Ni-alone (0.3 M and 1 M NaCl eluates) and Ni-BAF (0.3 M and 1 M NaCl eluates). For samples analyzed by iTRAQ (below), we used 100 µg Ni-BAF beads (final BAF concentration 1 µg/μl) and 5 mg nuclear lysate proteins.

### Liquid Chromatography-Coupled Tandem Mass Spectrometry

Eluted proteins were reduced and alkylated in solution using iodoacetamide, and proteolyzed with sequencing grade modified porcine trypsin (Promega, Madison, WI) as described [95]. Protein identification by LC-MS/MS analysis of peptides was performed with an LTQ ion trap MS (Thermo Fisher Scientific, San Jose, CA) interfaced with a 2D nanoLC system (Eksigent, Dublin, CA). Peptides were fractionated by reverse-phase HPLC on a 75 µm×100 mm C18 column with a 10 µm emitter using a 0–60% acetonitrile/0.5% formic acid gradient over 30 min at 300 nl/min. Peptide sequences were identified using Mascot software (version 2.2, Matrix Science, Boston, MA) to search the NCBI non-redundant database with the acquired fragmentation data. Identified sequences were confirmed manually by inspecting the fragmentation spectra. Scaffold (version Scaffold_2_04_00, Proteome Software Inc., Portland, OR) was used to validate MS/MS based peptide and protein identifications. Peptide identifications were accepted if they could be established at greater than 0% probability as specified by the Peptide Prophet algorithm [96] embedded within Scaffold. Low probability enabled potentially significant protein identifications based on many moderate-probability peptides, but no such proteins emerged; instead nearly all identifications were based on peptides with individual confidences >80% (Table S1). Protein identifications were accepted if they could be established at greater than 89% probability based on at least 1 identified peptide. Two “single-hit” candidates were subsequently validated as BAF-associated *in vivo*, as described in Results. Protein probabilities were assigned by the embedded Protein Prophet algorithm [97]. Proteins that contained similar peptides and could not be differentiated based on MS/MS analysis alone were grouped to satisfy the principles of parsimony.

### Isobaric Tagging for Relative and Absolute Quantitation

All four eluates (∼10 µg protein, each) were dissolved in 20 µl 0.1% SDS in 0.5 M triethylammonium bicarbonate, reduced, alkylated and digested at 37°C overnight with trypsin at a protein-to-enzyme ratio of 20∶1. The iTRAQ reagents (4-plex, Applied Biosystems - MDX Sciex, Foster City, CA) were dissolved in 70 µl ethanol, added to each digest, and incubated 1 hr at 22–25°C. The four samples, designated Ni-control-0.3 M eluate, BAF-0.3 M eluate, Ni-control-1 M eluate and BAF-1 M eluate, were labeled with iTRAQ reagents 114, 115, 116 and 117, respectively. After labeling, all samples were mixed and dried down to a volume of 50 µl. The combined peptide mixture was fractionated by Strong Cation Exchange chromatography (SCX) on a 1100 HPLC system (Agilent, Santa Clara, CA) using a PolySulfoethyl A column (2.1×100 mm, 5 µm, 300 Å, PolyLC, Columbia, MD), by first dissolving the sample in 4 mls SCX loading buffer (25% v/v acetonitrile, 10 mM KH_2_PO_4_ pH 2.8, adjusted with 1 N phosphoric acid), then loading the sample onto the column and washing isocratically for 30 min at 250 µl/min. Peptides were eluted by a gradient of 0–350 mM KCl (25% v/v acetonitrile, 10 mM KH_2_PO_4_ pH 2.8) over 40 min at a flow rate of 250 µl/min. The 214 nm absorbance was monitored and 10-SCX fractions were collected along the gradient. Each SCX fraction was dried down, dissolved in 40 µl 0.1% formic acid, and analyzed on a QSTAR Pulsar™ (Applied Biosystems - MDS Sciex, Foster City, CA) interfaced with an Eksigent nano-LC system (Dublin, CA). Peptides were separated on a reverse-phase column packed with 10 cm of C18 beads (360×75 µm, 5 µm, 120 Å, YMC ODS-AQ; Waters Milford, MA) with a 10 µm emitter tip (New Objective, Woburn, MA) attached. Peptides eluting from an HPLC gradient of 5–36% acetonitrile in 0.1% formic acid, over 60 min at a 300 nl/min flow rate, were sprayed into the QSTAR. Survey scans were acquired from *m/z* 350–1200 with up to three precursors selected for MS/MS using a dynamic exclusion of 45 sec. A rolling collision energy was used to promote fragmentation and the collision energy range was ∼20% higher than that used for unlabeled peptides due to iTRAQ tags.

MS/MS spectra were extracted and searched against the SwissProt database using ProteinPilot™ software (Applied Biosystems, Foster City, CA) with Paragon™ method and the following parameters: all species, trypsin as enzyme (one missed cleavage allowed), cysteine static modification with methylmethanethiosulfate and iTRAQ (peptide labeled at N-terminal and lysine) as sample type. Mass tolerance was set to 0.2 Da for precursor and 0.15 Da for fragment ions. The raw peptide identification results from Paragon™ Algorithm (Applied Biosystems, Foster City, CA) searches were further processed by the Pro Group™ Algorithm (Applied Biosystems, Foster City, CA) within the ProteinPilot™ software before they were displayed. The Pro Group™ Algorithm uses the peptide identification results to determine the minimal set of proteins that can be reported for a given protein confidence threshold. The peptide confidence threshold cutoff for the iTRAQ study was at least 95% (see Table 3).

### Protein expression plasmids, transfections and immunoprecipitations

FLAG-tagged constructs for the following proteins were used: MTA2 and Mi2β ([41]; provided by Y. Zhang, Univ. North Carolina, Chapel Hill), DPY-30-like ([42]; provided by K. Ge, NIH), Requiem (provided by E. M. Chan, Eli Lilly Corp.), human PARP1 ([37]; provided by W.L. Kraus, Cornell Univ.), CUL4A and DDB2 (provided by H. Wang, Univ. Alabama, Birmingham), GFP-tagged RBBP4 and RBBP7 (provided by T. Misteli, NIH). Each construct and its corresponding empty FLAG or GFP vector were transiently transfected into HeLa cells using LT1 (Mirus, Madison, WI) per manufacturer instructions and expressed for 24 hrs. Cells were then lysed by incubating 10 min on ice in immunoprecipitation (IP) buffer (20 mM HEPES pH 8, 300 mM NaCl, 0.3% Triton X-100, 0.2 mM EDTA, 0.2 mM EGTA, 1.5 mM MgCl_2_, 2 mM DTT, protease inhibitors, and benzonase [300 U/μl; EMD Chemicals Inc., Gibbstown, NJ]). Lysates were then diluted 1∶1 v/v with IP buffer to achieve final concentrations of 150 mM NaCl and 0.15% TX-100, incubated 10 more min on ice, sonicated 4 times (15 sec each) and centrifuged (50,000 rpm, 30 min, 4°C) to recover soluble proteins for subsequent immunoprecipitation. For Flag immunoprecipitations, 250 µl lysate was incubated 4 hrs (4°C) with 3 µl Flag-M2 agarose beads (Sigma, St. Louis, MO), then pelleted and washed 4 times with IP buffer. Bound proteins were eluted using either 2X-SB, or by two successive incubations (37°C, 10 min, each) with 100 µg/ml Flag-peptide (Sigma F4799; St. Louis, MO) when indicated. For GFP immunoprecipitations 250 µl lysate were incubated for 1 hr with 1 µl GFP antibody (Molecular Probes, A6455; Carlsbad, CA), after which 10 µl GammaBind G sepharose beads (Amersham, Piscataway, NJ) were added and incubation proceeded for 4 hrs more at 4°C. Beads were then pelleted and washed as above, and proteins were eluted with 2X-SB, resolved by SDS-PAGE (4–12% NuPAGE™ gels; Invitrogen Carlsbad, CA) and Western blotted with the following antibodies: rabbit anti-Flag (1∶7000 dilution; Sigma F7425; St. Louis, MO), mouse anti-GFP (1∶5,000; Santa Cruz Biotechnologies, sc-9996; Santa Cruz, CA), rabbit anti-Emerin (1∶2,000; Santa Cruz Biotechnologies, sc-15378; Santa Cruz, CA), rabbit anti-Lamin A/C (1∶2,000; Santa Cruz Biotechnologies, sc-20681; Santa Cruz, CA), rabbit anti-DDB1 (1∶1,000; Abcam 21080; Cambridge, MA), mouse anti-PARP1 (1∶5,000; BD Pharmingen 556362; San Jose, CA), mouse anti-γ-tubulin (1∶500,000; Sigma T6557; St. Louis, MO) and rabbit serum 3273 against BAF (1∶10,000 dilution; [15]). Western blots were visualized by horseradish peroxidase chemiluminescence (Amersham, Piscataway, NJ).

### UV irradiation of transiently transfected HeLa cells

About 22–24 hrs after transfection cells were UV-irradiated (20 J/m^2^) using a Stratagene UV Stratalinker 2400 (Stratagene, La Jolla, CA), given fresh media and allowed to recover for the indicated time, then harvested with a rubber policeman, washed once with ice-cold PBS, pelleted (1,500 rpm, 5 min, 4°C) and processed for immunoprecipitation as described above.

## Supporting Information

Table S1 Excel spreadsheet showing peptides used to identify proteins with at least 89% confidence, as determined by Scaffold analysis of LC-MS/MS results for each eluate (0.3 M no BAF, 0.3 M BAF, 1 M no BAF, 1 M BAF).
(1.53 MB XLS)
